# Prevalence of drifting osteons distinguishes human bone

**DOI:** 10.1371/journal.pone.0298029

**Published:** 2024-02-23

**Authors:** Katherine M. French, Sophia R. Mavroudas, Victoria M. Dominguez

**Affiliations:** 1 School of History, Archaeology & Religion, Cardiff University, Cardiff, United Kingdom; 2 Department of Anthropology, Washington State University, Pullman, Washington, United States of America; 3 Forensic Anthropology Center at Texas State, Department of Anthropology, Texas State University, San Marcos, Texas, United States of America; 4 Department of Anthropology, Lehman College-CUNY, Bronx, New York, United States of America; 5 Department of Anthropology, The Graduate Center-CUNY, New York, New York, United States of America; 6 New York Consortium in Evolutionary Primatology, New York, New York, United States of America; University of Gothenburg: Goteborgs Universitet, SWEDEN

## Abstract

The histological, or microscopic, appearance of bone tissue has long been studied to identify species-specific traits. There are several known histological characteristics to discriminate animal bone from human, but currently no histological characteristic that has been consistently identified in human bone exclusive to other mammals. The drifting osteon is a rare morphotype found in human long bones and observationally is typically absent from common mammalian domesticates. We surveyed previously prepared undecalcified histological sections from 25 species (human *n* = 221; nonhuman primate *n* = 24; nonprimate *n* = 169) to see if 1) drifting osteons were indeed more common in humans and 2) this could be a discriminating factor to identify human bone histologically. We conclude that drifting osteons are indeed more prevalent in human and nonhuman primate bone relative to nonprimate mammalian bone. Two criteria identify a rib or long bone fragment as human, assuming the fragment is unlikely to be from a nonhuman primate given the archaeological context: 1) at least two drifting osteons are present in the cross-section and 2) a drifting osteon prevalence (or as a percentage of total secondary osteons) of ≥ 1%. We present a quantitative histological method that can positively discriminate human bone from nonprimate mammalian bone in archaeological contexts.

## Introduction

**Fig 1:**
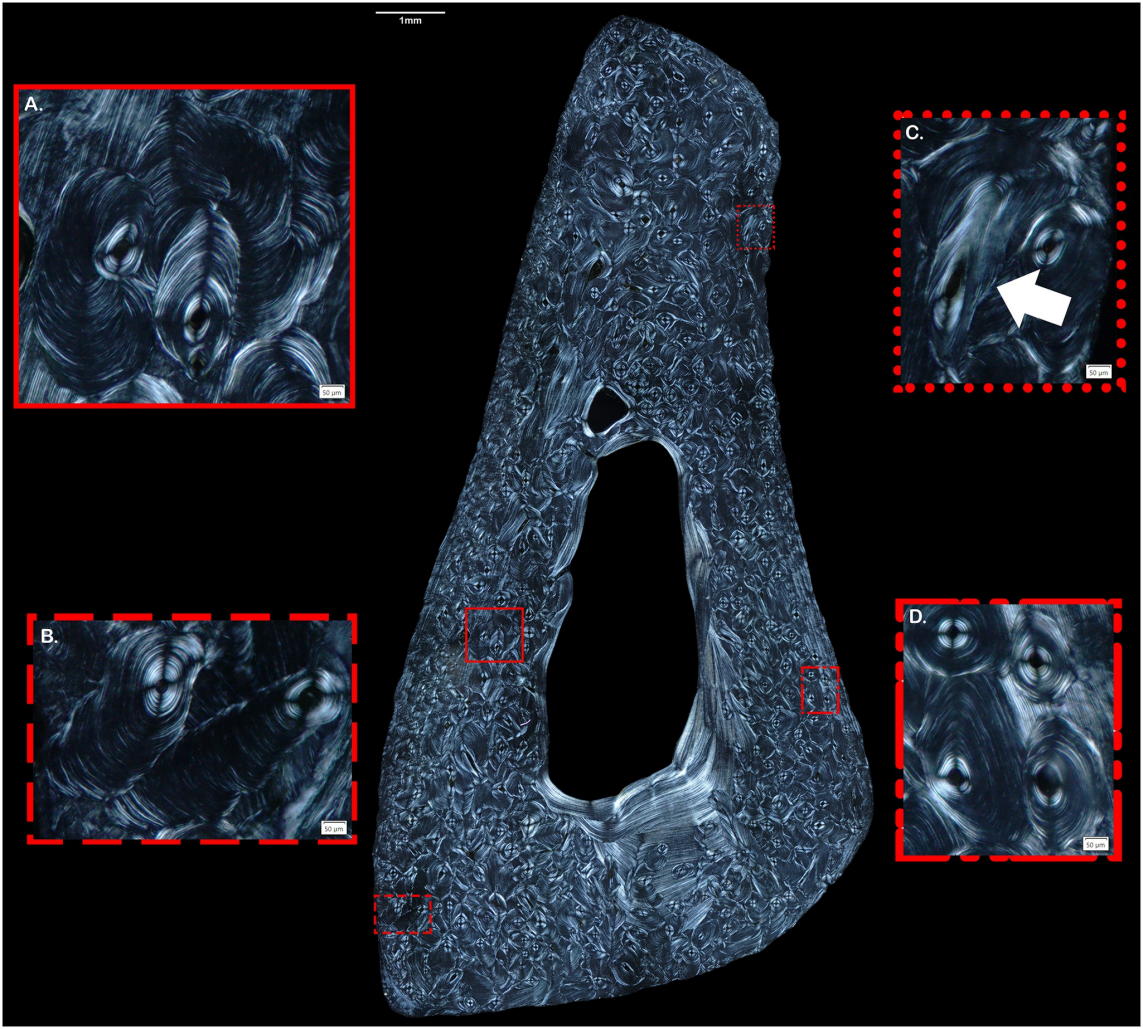
Osteon morphotype examples from a 24-year-old human male fibula. A) Two DOs with lamellae on an irregular pathway. B) DO lamellae following a linear path with the lamellae forming towards the periosteum. C) Arrow indicates an oblique osteonal canal representing an oblique cross-sectional cut that should not be confused with a DO. D) Examples of typical secondary osteon morphotype with no evidence of drift.

Drifting osteons (DOs) are a secondary osteon morphotype formed when a developing osteon develops transversely through cortical bone while forming longitudinally (Fig 1). In cross-section, they appear as a hemicyclical tail of lamellae trailing the osteonal canal [1]. The transverse appearance of a DO’s lamellae can be either a straight line or on an irregular pathway, but the Haversian canal is consistently closer to the endosteal surface and the trailing lamellae are progressively formed towards the periosteal surface, which is the area of more rapid growth (Fig 1A and 1B) [1–3]. The etiology of this morphotype is unknown, although both biomechanical and metabolic explanations have been suggested [4–6]. Prior research has also demonstrated that DOs are more prevalent in juvenile bone [7, 8].

The prevalence of DOs has received limited discussion as a discriminating factor between human and nonhuman mammalian bone [9–11]. When differentiating human from nonhuman bone, histologists generally focus on the presence or absence of key histological structures such as primary fibrolamellar plexiform bone or osteon banding [e.g. 12–17]. Alternatives to qualitative observation have been explored more recently, including histomorphometric variation to differentiate human from nonhuman bone [18–21].

Although both histomorphological and histomorphometric methods are successfully employed in practice, neither the qualitative nor quantitative markers available can definitively differentiate between human and nonhuman bone one hundred percent of the time. Bone histologists have anecdotally discussed the presence and abundance of drifting osteons as an additional distinguishing marker between human and nonhuman bone, with some research suggesting that mammalian cortical bone can be differentiated using drifting osteons [11], though this has not yet been quantified. There has been limited discussion in the literature to date as to the presence of drifting osteons in nonhuman mammal bone specifically [10, 11], and the relative abundance of drifting osteons in nonhuman mammals compared to humans requires further study.

This study quantifies the prevalence of DOs within a sample of mammalian species to explore the potential for this variable to discriminate human from nonhuman bone in archaeological assemblages. Our results demonstrate that the prevalence of DOs is the only histomorphological characteristic at present that can positively distinguish human bone from nonprimate mammalian bone.

## Materials and methods

This study incorporates 314 (human *n* = 221; nonhuman primate *n* = 24; nonprimate *n* = 169) undecalcified histological bone samples from archaeological, forensic, and paleontological contexts across 25 different mammal species (including nonhuman primates) from existing collections in North America (Table 1, S1 Table). It is possible that multiple samples came from one individual but different skeletal elements (S2 Table). The sample includes a wide variety of extant and extinct species found commonly in both archaeological and forensic contexts spanning across the United States and Europe. Since the practical application of human versus nonhuman bone differentiation can include fragments from any anatomical position, a variety of long bone, rib, mandible and cranial elements were used. However, the osteogenesis of cranial and facial elements is distinct from long bones and it is unknown if the prevalence of drifting osteons may be similarly distinct.

**Table 1:** Species, element, and age category information for the study sample.

Species	Family	Common Name	Elements	Juvenile	Adult	Unknown	Total
*Bison bison*	Bovidae	American bison	Rib	0	1	0	1
*Bos primigenius*	Bovidae	Cow	Femur, Metatarsal, Radius, Rib, Tibia	0	11	8	19
*Bubalus Bubalis*	Bovidae	Water Buffalo	Femur, Radius	0	0	3	3
*Canis sp*.	Canidae	Canine (unspecified)	Femur, Humerus, Mandible, Rib, Temporal	0	4	25	29
*Capra hircus*	Bovidae	Goat	Femur, Mandible, Metapodial, Tibia	0	0	4	4
*Cervidae sp*.	Cervidae	Deer (unspecified)	Femur, Humerus, Radius, Rib, Tibia	11	9	21	41
*Didelphimorphia sp*.	Didelphidae	Opposum	Femur, Tibia	2	1	0	3
*Dinictis sp*.	Nimravidae	False saber-toothed cat (extinct)	Rib	0	0	1	1
*Equus caballus*	Equidae	Horse	Femur	2	15	4	21
*Felis catus*	Felidae	Domestic cat	Femur, Humerus, Mandible, Rib, Tibia	0	3	4	7
*Herpestidae sp*.	Herpestidae	Mongoose	Femur	0	0	1	1
*Homo sapiens*	Hominidae	Human	Femur, Fibula, Metatarsal, Rib	32	189	0	221
*Lepus sp*.	Leporidae	Hare/rabbit (unspecified)	Rib, Temporal	0	0	2	2
*Mustela sp*.	Mustelidae	Mustelid (unspecified)	Rib	0	0	1	1
Primate†	Unknown	Primate (unspecified)	Femur, Humerus, Tibia	0	0	14	14
*Odocoileus virginianus*	Cervidae	White-tailed deer	Femur, Humerus	0	0	2	2
*Oxyaena ultima*	Oxyaenidae	Hyena (extinct)	Long Bone (not specified)	0	0	1	1
*Pan troglodytes*	Hominidae	Chimpanzee	Femur, Humerus	0	0	10	10
*Panthera leo*	Felidae	Lion	Femur	0	0	1	1
*Paradoxurus hermaphroditus*	Viverridae	Asian palm civet	Femur, Rib	0	0	2	2
*Peccary sp*.	Tayassuidae	Peccary (unspecified)	Rib	0	0	1	1
*Procyon lotor*	Procyonidae	Raccoon	Humerus, Tibia	0	3	0	3
*Sus sp*.	Suidae	Pig (unspecified)	Femur, Fibula, Humerus, Rib, Tibia	1	0	20	21
*Taxidea taxus*	Mustelidae	American badger	Mandible	0	0	1	1
*Testudines sp*.	Humerus	Turtle (unspecified)	Long Bone (not specified)	0	2	0	2
*Ursidae sp*.	Ursidae	Bear (unspecified)	Fibula, Rib	1	0	1	2

^†^Available documentation for this sample indicates the slides were from various primate species, none of which were great apes.

Histological slides were evaluated using light microscopy at 10x magnification in bright field or linear polarized light, depending on collagen preservation. One slide per bone was analyzed including partial and whole cross-sections, and 100% of the available cross-section was analyzed. Partial cross-sections included a minimum of 10% of the cross-sectional area of the bone. Images were captured using either CellSens or Leica DM6M Scope Software and uploaded to ImageJ for quantitative analysis when necessary.

**Fig 2:**
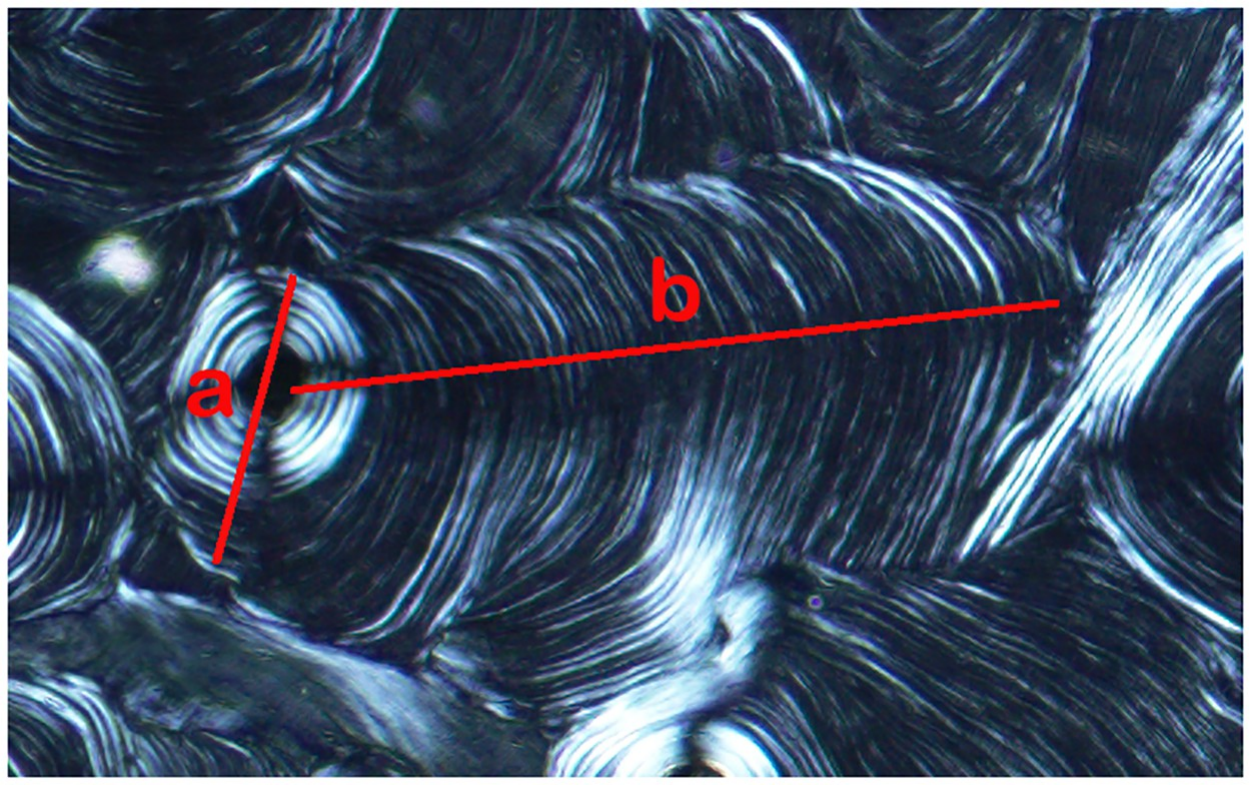
Drifting osteon as defined in this study. A DO must have a circular Haversian canal and possess a hemicyclical tail (b) that is at least twice the length of the maximum diameter of the osteon (a). This image illustrates a linear DO.

Data collection proceeded in two stages. The first was a general survey to determine whether drifting osteons are present across a range of species. The following information was collected for each slide: species, skeletal element, skeletal age (adult, juvenile, or unknown), and number of DOs present. Species was identified based on specimen labels and available collection notes. Skeletal age was evaluated based on complete fusion of long bone elements when possible—fused elements were recorded as adult and unfused as juvenile. To ensure consistency between analysts, DOs were strictly defined as any transversely drifting secondary osteon with a circular Haversian canal observed in a transverse cross-section in which the length of the hemicyclical tail is at least twice the maximum diameter of the osteon (Fig 2).

In the second stage of data collection, additional information was collected in order to determine what the overall prevalence of DOs was in a micrograph sample as a percentage of the total number of secondary osteons. This proceeded with a smaller subset of the sample that was available for follow-up analysis (human *n =* 124; nonprimate *n =* 81), and the numbers of intact and fragmentary osteons were recorded. An intact osteon is defined as having a complete Haversian canal and reversal line, whereas a fragmentary osteon has its Haversian canal totally or partially obliterated by an adjacent osteon or resorption bay [22]. To account for differences in sampling sites, cortical area, and allometry, the prevalence of DOs was calculated as a percentage of total osteons observed in a transverse cross-section as follows:

$$\text{DO}\;\text{Prevalence}=\frac{\text{Number}\;\text{of}\;\text{DOs}}{\text{Total}\;\text{of}\;\text{secondary}\;\text{osteons}\;\left(\text{DOs}\;,\;\text{Intact,}\;\;\text{and}\;\text{Fragmentary}\right)}$$

DO count and prevalence ratios were then compared between broad taxonomic groups (human, nonprimate) and age cohorts (juvenile, adult). All data are available in S2 Table.

## Results

Table 2 shows the summary statistics for the number of DOs identified per individual within each broad taxonomic classification group and age categories. Results demonstrate that the DO morphotype is far rarer in nonprimate mammals (${\bar{\text{x}}}_{\text{DO}\;\text{count}}=0.107$) compared to humans (${\bar{\text{x}}}_{\text{DO}\;\text{count}}=3.412$) (Table 2). Further, DOs were identified in only 9 of 169 (5.3%) of the nonprimate samples as compared with 173 of 245 (70.6%) primate samples, humans included. Nonprimate species with identified DOs include *Canis familiaris* (*n* = 4), *Sus sp*. (*n* = 1), *Lepus californicus* (*n* = 1), extinct *Oxyaena ultimo* (*n* = 1), *Ursidae sp*. (*n* = 1), and *Panthera leo* (*n* = 1).

**Table 2:** Summary statistics, DO counts per individual in human, nonhuman primate and nonprimate groups.

Group	*N*	Mean	Std. Deviation	Min, Max	Median
*Overall*					
Humans	221	**3.412**	5.56	0, 50	2
Nonhuman Primate	24	**2.625**	2.96	0, 9	2
Non-Primates	169	**0.107**	0.50	0, 4	0
*Humans*					
Juvenile	97	**2.688**	2.49	0, 8	2
Adult	124	**3.534**	5.92	0, 50	2
*Non-Primates*					
Juvenile	17	**0.176**	0.73	0, 3	0
Adult	49	**0.041**	0.29	0, 2	0

**Fig 3:**
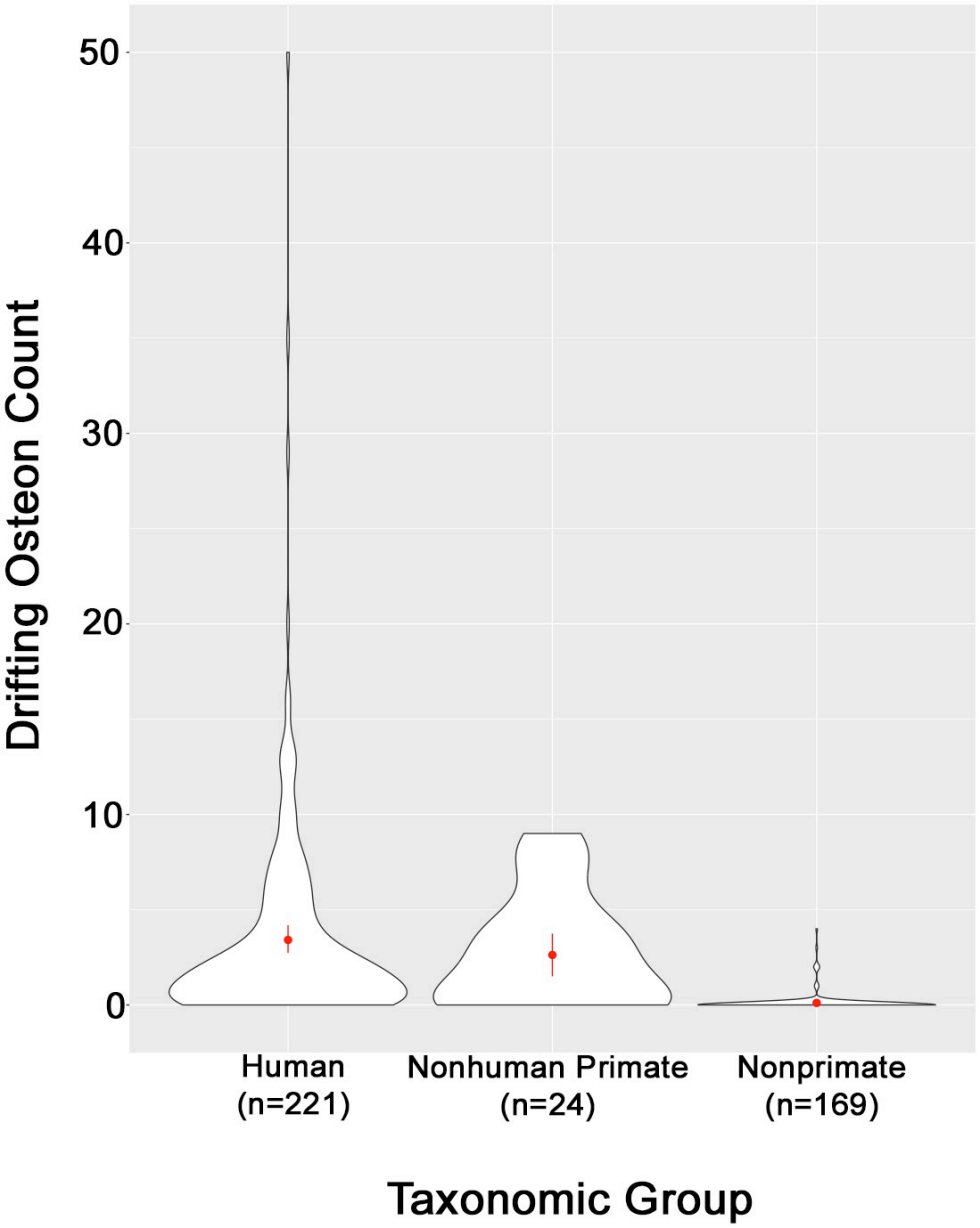
Violin plot showing the distribution of DO counts for each taxonomic group.

The nonhuman primate sample was limited in size (*n* = 24) and data quality. Prevalence percentages were not calculated for nonhuman primates due to lack of data for intact and fragmentary osteon counts. Nonhuman primates are also not included in any assessment of age as a discriminating variable because the entire available sample was of unknown age. Our results show that mean DO count is higher amongst humans compared to nonhuman primates, but given the high standard deviation of the human sample and the substantial overlap between the two groups, the prevalence of DOs is unlikely to be a robust discriminating factor between human and nonhuman primates (Fig 3).

**Fig 4:**
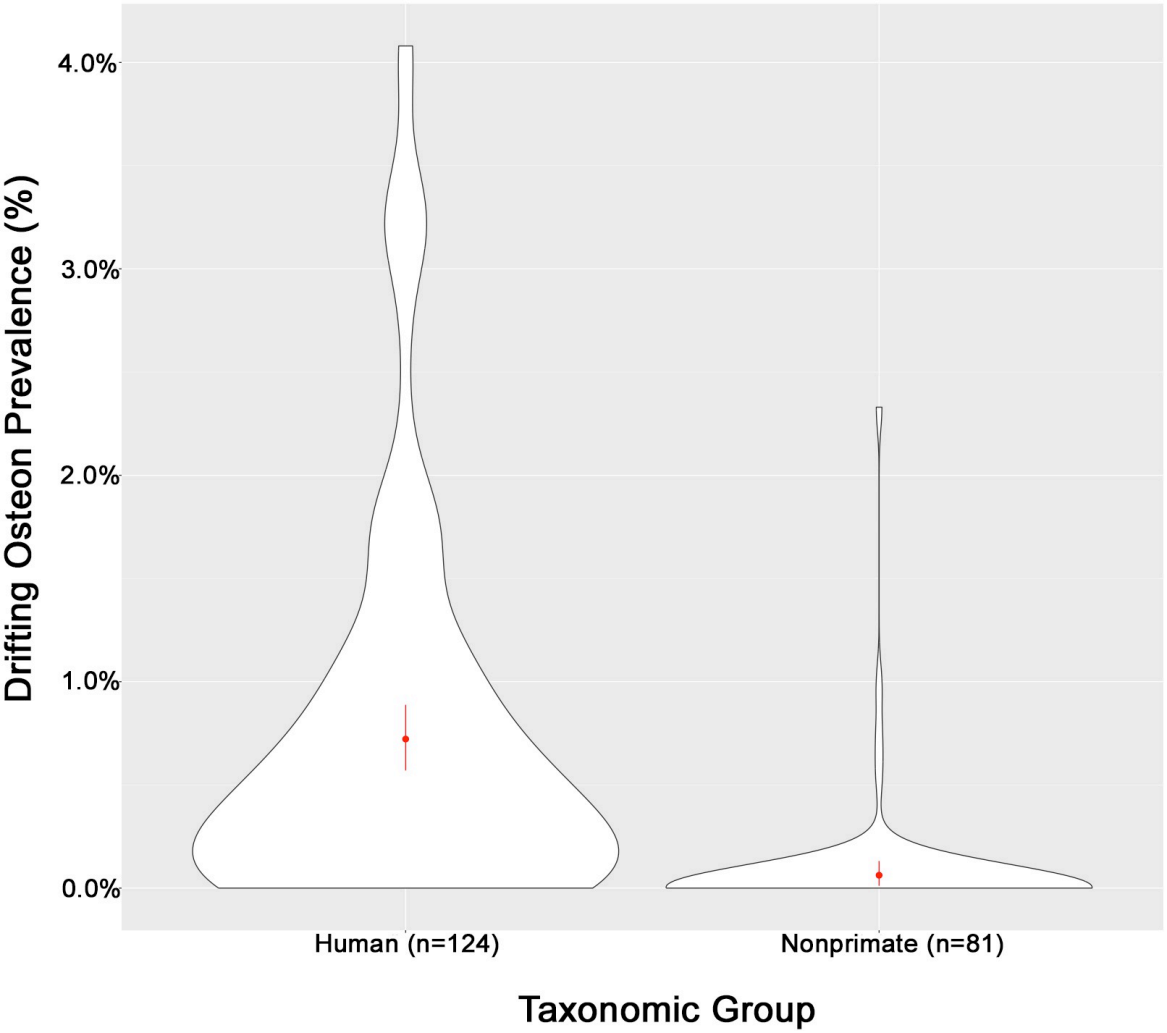
Violin plot showing the distribution of DO prevalence comparing each taxonomic group.

Prevalence of DOs should be a more robust variable for comparison between taxonomic or age groups, as DO count is reported in relation to the total number of secondary osteons. This provides a useful correction for relative age, remodeling rates, cortical area, and allometry. The human prevalence rate across all age categories is 0.72% (Table 3). This confirmed that overall, the DO is a rare morphotype of secondary osteon. DOs are substantially rarer amongst nonprimate individuals. While there are a few positively skewed outliers, nonprimate prevalence of DOs is typically 0%, with the mean for the sample just above at 0.06% (Table 3, Fig 4).

**Table 3:** Summary statistics, DO prevalence data for individuals in human and nonprimate groups.

Group	*N*	Mean	Std. Deviation	Min, Max	Median
*Overall*					
Humans	124	**0.72%**	0.91	0%, 4.08%	0.39%
Non-Primates	81	**0.06%**	0.29	0%, 2.33%	0.00%
*Humans*					
Juvenile	1	**-**	-	0.27%	-
Adult	123	**0.73%**	0.01	0%, 4.08%	0.40%
*Nonprimate*					
Juvenile	16	**0.05%**	0.18	0%, 0.73%	0.00%
Adult	48	**0.00%**	7.22E-05	0%, 0.05%	0.00%
Unknown	17	**0.25%**	0.59	0%, 2.33%	0.00%
*Age Groups* †					
All Juveniles	17	**0.06%**	0.18	0%, 0.73%	0.00%
All Adults	171	**0.52%**	0.01	0%, 4.08%	0.20%

^†^Nonhumans are overrepresented in the juvenile group; humans are overrepresented in adult group.

Skeletal element type and age category variables were also observed to determine impact on DO frequency. Nearly the entire sample across taxonomic groups consisted of rib and long bone fragments (*n =* 414). The nonprimate sample included a limited number of mandible and cranial fragments (*n* = 7), which therefore did not allow for a robust comparison with long bones or ribs. Six of the samples did not contain a DO. One sample from the zygomatic arch of a lagomorph had the highest DO prevalence of all nonprimate samples (2.33%), a clear outlier within the nonprimate group (Fig 4). In terms of age categories, DO counts are higher in adult humans (${\bar{\text{x}}}_{\text{DO}\;\text{count}}=3.53$) than juvenile humans (${\bar{\text{x}}}_{\text{DO}\;\text{count}}=2.69$); however, we were unable to calculate the mean prevalence for juvenile humans (Tables 2 and 3). There was a slight difference between age categories in the nonprimate groups (Tables 2 and 3), but overall, the values were low across nonprimate groups.

## Discussion and conclusions

### Application for human bone identification

These data support previously reported observations that DOs are much more common in humans than nonprimate mammals. How then can this observed difference be applied to the histological identification of bone from archaeological sites that cannot morphologically be distinguished as human or nonhuman? The simplest method is to identify within our data the cutoff point above which the sample must be primate. Prevalence rather than DO count should be used, as it controls for potential species or sample differences in fragment pattern, cortical area, and allometry. Based on our findings, the cutoff is a 1% DO prevalence, above which the sample is almost certainly primate. A 1% DO prevalence could otherwise be stated as a probability that out of 100 secondary osteons sampled, one will be a drifting osteon.

In our sample, only one nonprimate, *Lepus californicus*, would incorrectly be identified as human using a 1% DO prevalence rate as a cutoff. There are two issues with this sample that may explain its outlier status. First, lagomorphs are notoriously difficult to classify histologically, particularly using standard histomorphological characteristics. For example, they are one of the only nonhuman mammalian species to not develop primary fibrolamellar plexiform bone [23]. Second, the sample is of a zygomatic arch rather than a long bone fragment as is the majority of the sample. Our results suggest there may be a difference in DO prevalence between long bone and cranial elements, and further exploration with a larger sample size is needed to confirm these findings. The only other nonprimate sample with a DO prevalence approaching 1% is from the extinct Lower Eocene carnivore *Oxyaena ultimo*, an unlikely contributor to archaeological assemblages.

**Fig 5:**
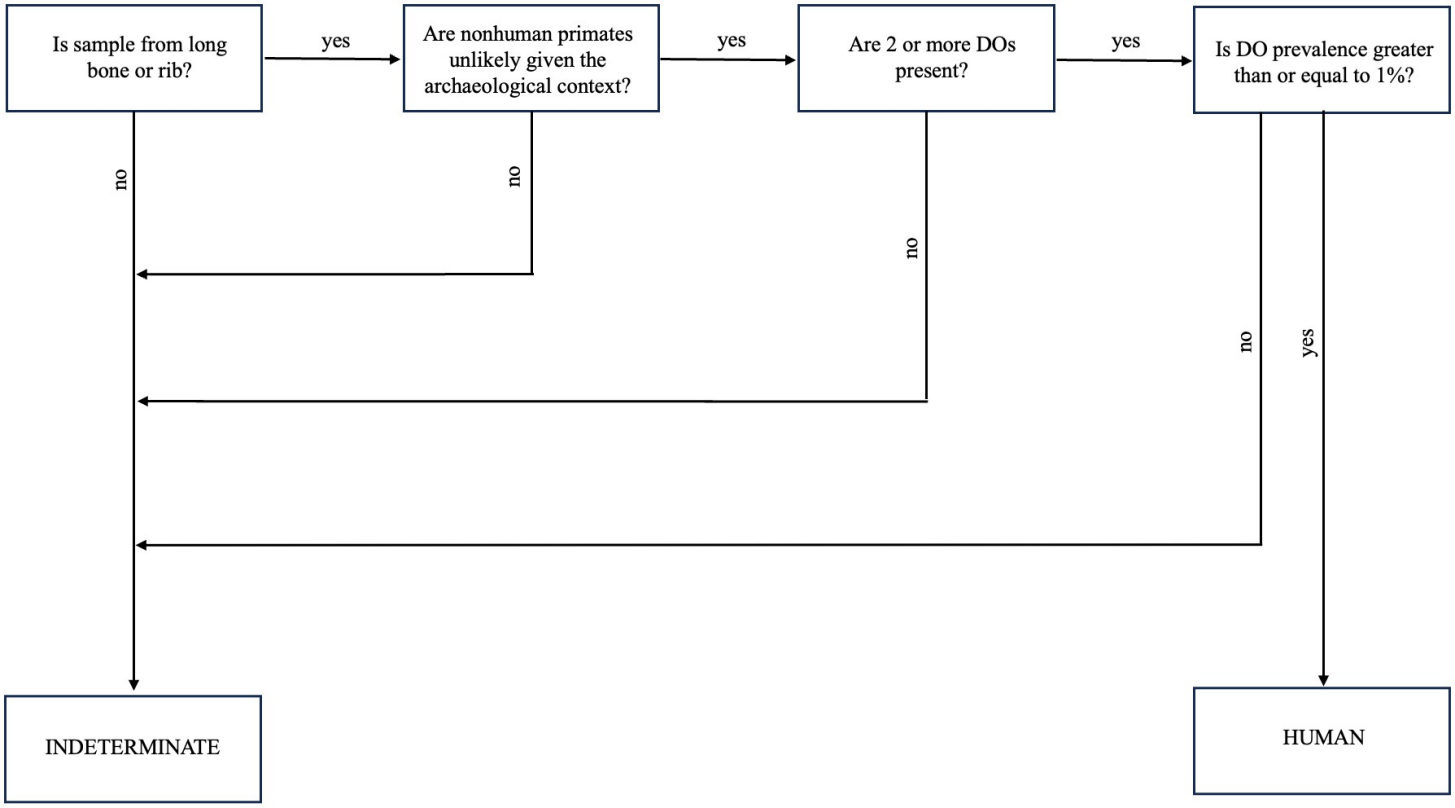
Decision tree illustrating how to use the recommended criteria to identify human bone.

Quantifying the prevalence of drifting osteons can therefore be a useful tool for discriminating human bone fragments. We recommend fragments submitted for this analysis should be from a rib or long bone with a minimum 10% of the cross-sectional area present and the sample is highly unlikely to be from a nonhuman primate given the archaeological context. If the sample is poorly preserved (OHI of <5, then a greater cross-sectional area will be required so that the equivalent of 10% of the total cross-sectional area of the slide is readable. We recommend the following DO criteria are met for a positive identification of human bone: 1) a DO count ≥ 2 and 2) a DO prevalence of ≥ 1% (Fig 5). If these conditions hold, a bone fragment is almost certainly human. Following this method would avoid identifying any nonprimates as human; however, it is also conservative. Only 31 of 124 (25%) humans in our sample would be positively identified as human, but nonhuman fragments would not be incorrectly classified as human.

The power of this method is amplified when combined with long-established histomorphological methods for human/nonhuman differentiation, particularly the identification of fibrolamellar plexiform bone and osteon banding in nonhuman bone. The value of documenting the prevalence of drifting osteons is to classify bone fragments as human, but these other well-documented characteristics classify bone fragments as nonhuman. Fibrolamellar plexiform bone is the main primary bone type in large fast-growing mammals. It develops as a fibrous bone scaffolding with later-forming lamellar bone enclosing a web-like vascular plexus that radiates in all directions, often described as having a “brick-like” appearance [24, 25]. Examples of fibrolamellar plexiform bone have been reported in human infants, but are no longer present after the first year of age [26, 27]. This is in stark contrast to most nonprimate mammal bone where large swaths of plexiform bone are present into skeletal maturity and are easily distinguishable from human primary lamellar bone. Osteon banding appears in cross-section as five primary or secondary osteons in a row. While Mulhern and Ubelaker [28] mention observing osteon banding in humans, they point out that it is still useful for distinguishing human and nonhuman bone in applied contexts since human banding is isolated, whereas nonhuman banding generally occurs within plexiform bone or is stacked in several parallel rows. Limited osteon banding has been found in studies of human bone [29, 30]; however, multiple bands of osteons combined with a general linear primary bone structure or coupled with the presence of fibrolamellar plexiform bone remains indicative of nonhuman bone.

The presence of fibrolamellar plexiform bone is therefore the most useful determining factor for identifying younger nonhuman animals that will have transverse cross-sections showing this primary bone structure and very few secondary osteons. Osteon banding is also more common in younger nonhuman individuals, where significant remodeling has not yet obliterated the banded pattern. The limitations of these methods are therefore in identifying nonhumans of advanced age with significant remodeling and positively identifying human bone. Using these methods in conjunction, therefore, will provide the best and most comprehensive results for differentiating human from nonprimate bone.

Other histomorphometric methods such as osteon size and circularity have been evaluated in some limited mammal species with the goal of differentiating human from nonhuman bone in an applied context [18–21]. These studies indicate that human osteons are more variable in size than nonhuman osteons, and there are conflicting reports as to whether human or nonhuman osteons are more circular [31]. Further research on histomorphometric species differentiation, including morphometric analysis of DOs that are often excluded from such studies [e.g. 31], will refine its utility and has the potential to accurately differentiate between nonhuman species.

### Effect of age on DO prevalence

DOs have been posited as a distinguishing feature between human adult and juvenile bone [7, 8]. Research indicates an inverse relationship between age and the presence of DOs, with younger individuals having a higher prevalence of DOs [32–34], although DOs are still documented in adult human bone [5, 6, 35, 36]. Streeter [37] published a method of aging human juvenile ribs characterizing the presence of linear DOs as indicative of bone from adolescents. Therefore, age is a key variable to consider in any study of DO prevalence.

**Fig 6:**
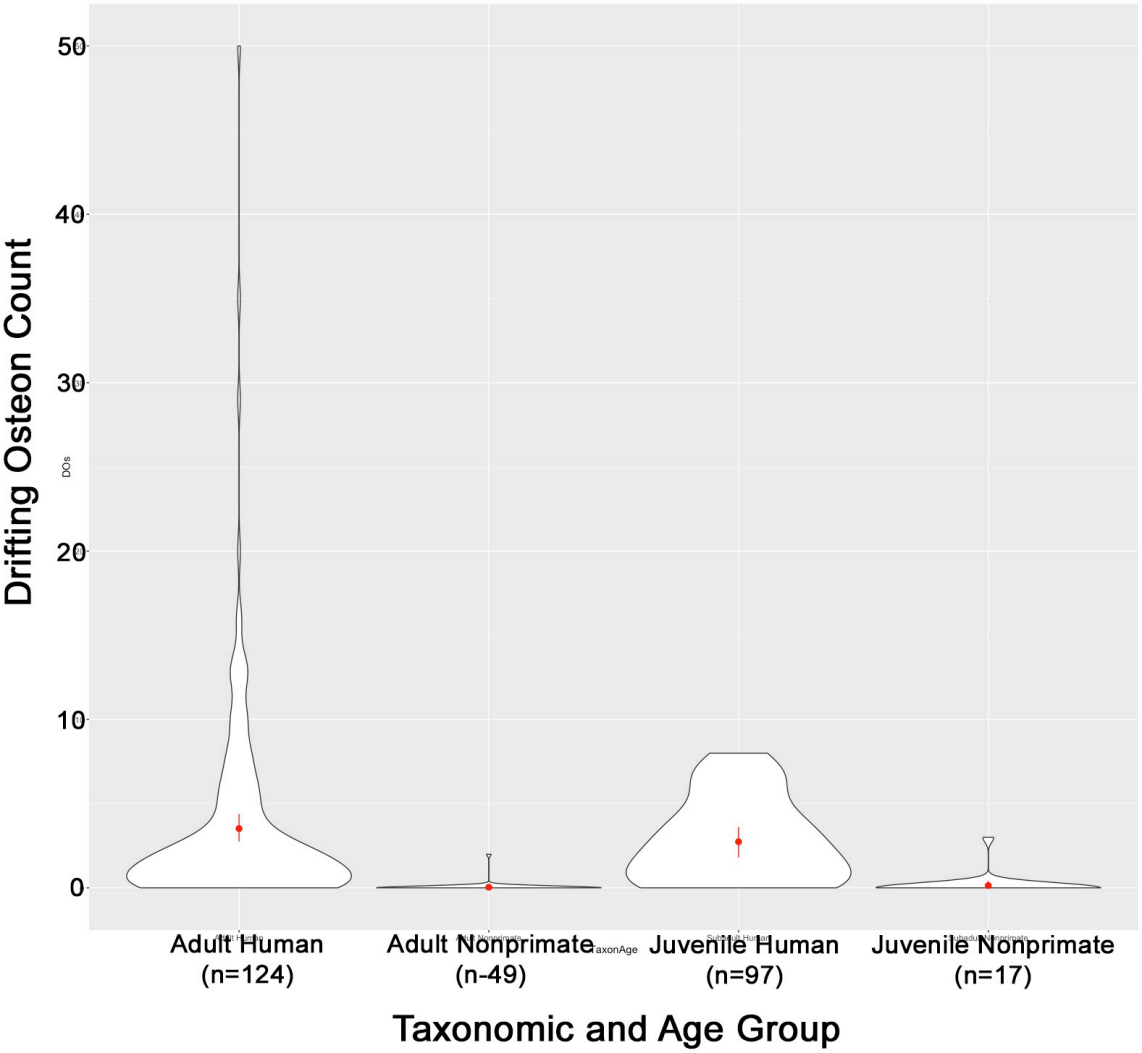
Violin plot showing the distribution of DO count for human and nonprimate groups in both age categories.

Our findings show that membership in the human taxonomic group is a stronger predictor of DO count than age category (Fig 6). Further, contra to previous studies [e.g. 32, 33, 37], we did not find that juvenile humans or nonhumans had a significantly higher count or prevalence of DOs than adult groups. However, our “adult” category was broad, obscuring nuance in different age categories. Recent studies have found DOs highest in the 20- to 30-year age group, classified as “adult” in our study, but that DO osteon population density (OPD) decreased after age 30 [34, 36]. The increase in DO OPD in juvenile and young adult humans may therefore be attributable to growth and development of peak bone mass, which, once achieved, tends to decline steadily into old age. However, DOs are still identified in humans into their nineties [36], so this method may still positively identify humans in any age category.

### Etiology of drifting osteons

What are the potential explanations for the higher occurrence of drifting osteons in human bone? Maggiano et al. [38] demonstrate that remodeling preferentially follows existing vascular canals. The three-dimensional vascular network present in fibrolamellar plexiform bone therefore provides multiple avenues for microcrack repair or other targeted remodeling events. Human bone, on the other hand, has more limited primary vascular pathways, which might necessitate a bone modeling unit (BMU) to “drift” transversely through the cortical space to access areas for targeted remodeling. Andronowski and Cole [39] refer to this as the transverse drift of an osteon “steering” towards a microcrack to repair. Mechanisms for such lateral motion have been proposed previously [40]. Alternatively, the high diversity of secondary osteon morphotypes in primate bone, including DOs, might reflect greater vascular plasticity, allowing for a broader potential variation in vascular connectivity within primate bone relative to nonprimate mammals [41].

Further research is needed to clarify the etiology and function of the drifting osteon morphotype, which may further elucidate the observed taxonomic and age-related differences in DO prevalence. Biomechanical proponents suggest DOs are a result of varying strain placed on the cortex of bone [42]. Strain on the microscopic level is more varied than on the gross level, which may help explain why strain forces cause DOs to form in the same cortex and at the same time as longitudinal secondary osteons [43]. Proponents of metabolic causes postulate that DOs are a consequence of the body utilizing mineral stores within bone at times of stress [44]. However, Epker and Frost [2] discovered no difference in DO frequency between males and females in any age category, which supports a non-metabolic cause for DOs since females in advanced decades typically require more calcium stores with progressing age [45].

### Future research directions

Our preliminary findings suggest that the DO prevalence method is unlikely to be a robust metric to differentiate human from nonhuman primate bone (Fig 3), so the method should not be used in contexts where there is potential for nonhuman primate bone as mentioned in Criterion 2 (Fig 5). Follow up research with a larger nonhuman primate sample that controls for age may provide a more nuanced perspective.

Every attempt was made to control for age in our analysis; however, the lack of consistent aging data was a limiting factor. Age data was not available for 103 of 169, or 61% of nonhuman samples. DO prevalence and age could only be evaluated using the broadest age categories of adult versus juvenile. An increased prevalence of DOs has never been documented in nonprimate mammalian juveniles, although this may be due to a lack of research. Further we did not have sufficient data to calculate DO prevalence for the juvenile human group.

As was the case with fibrolamellar plexiform bone and osteon banding [27, 30, 39], it is possible that exceptions will be found within age categories, skeletal elements, or biomechanical outliers in nonhuman bone that may have a higher prevalence of drifting osteons than reported in this study. Additional research is warranted looking at DOs in different age categories of nonhuman mammalian bone, which has received limited attention to date. Similarly, our study was limited in looking at only a few instances of cranial fragments or other flat bones. As reported earlier, the main nonhuman outlier was from a zygomatic arch, supporting the need for further study of all skeletal elements, not only ribs and long bones. It will also be important to confirm using a larger dataset that the prevalence of drifting osteons is comparable across all long bone and rib categories, controlling for age and species.

In summary, this research presents the first method for the histological identification of human bone in applied contexts. In contexts where DNA or proteins are not preserved, such as cremations or tropical environments, this is currently the *only* method to positively differentiate human from nonprimate bone fragments that cannot be distinguished morphologically. We welcome a blind validation study testing the proposed method using a novel, known-species dataset. Future research is encouraged exploring the addition of additional flat, cranial, and facial skeletal elements and an increased diversity of species to ensure this method can be applied outside of North American contexts.

## Supporting information

S1 Table Details of histological collections used in this study. (XLSX)

S2 Table Collection of origin and known information for each slide included in the study. (XLSX)

S1 File Inclusivity in global research. (DOCX)
